# Impact of SARS-CoV-2 infection and viral burden on outcomes in acute decompensated heart failure

**DOI:** 10.1186/s12879-026-14407-y

**Published:** 2026-09-18

**Authors:** Mohamad Amer Nashtar, Haris Sehovic, Mohammed Salemdawod, Gizem Garipoglu, Betül Ödemis, Asterios Tzalavras, Martin Steinmetz, Ali Canbay, Mustafa Özçürümez, Varnavas Varnavas, Polykarpos Christos Patsalis, Antonios Katsounas

**Affiliations:** 1 Ruhr University Bochum, Knappschaft Kliniken University Hospital Bochum, Department of Medicine, Bochum, Germany; 2 Division of Cardiology, Department of Cardiovascular Diseases, Cliniques Universitaires St. Luc, Brussels, Belgium

**Keywords:** Acute decompensated heart failure, COVID-19, SARS-CoV-2, Viral load, Cycle threshold, Intensive care unit, In-hospital mortality, Heart failure outcomes

## Abstract

**Background/Objectives:**

Patients hospitalized with acute decompensated heart failure (ADHF) represent a clinically vulnerable population. However, the impact of concomitant severe acute respiratory syndrome coronavirus 2 (SARS-CoV-2) infection and the prognostic relevance of viral burden in this setting remain incompletely understood.

**Methods:**

We conducted a retrospective cohort study including 2,002 adults hospitalized with ADHF at a tertiary academic center between March 2020 and December 2024. SARS-CoV-2 infection was confirmed by reverse transcription quantitative polymerase chain reaction (RT-qPCR) during the index hospitalization. The primary outcome was in-hospital mortality. Secondary clinical outcomes were ICU admission and invasive mechanical ventilation, while hospital and ICU length of stay were assessed as resource-utilization outcomes. Multivariable regression models adjusted for clinically relevant covariates were used to assess associations between infection status and outcomes. Among infected patients, cycle threshold (Ct) values were analyzed as surrogate markers of viral burden.

**Results:**

Of the 2,002 patients included, 246 (12.3%) had confirmed SARS-CoV-2 infection. Compared with uninfected patients, infected individuals had higher in-hospital mortality (32.5% vs. 20.6%), ICU admission (61.4% vs. 50.7%), and invasive mechanical ventilation (6.5% vs. 2.4%) (all *p* ≤ 0.002). After multivariable adjustment, SARS-CoV-2 infection remained associated with in-hospital mortality (aOR 2.63, *p* = 0.0002), ICU admission (aOR 1.79, *p* = 0.0027), and invasive mechanical ventilation (aOR 2.84, *p* = 0.0017). Infection was also associated with longer hospital stay (aβ + 9.23 days, *p* < 0.0001) and longer ICU stay (aβ + 7.85 days, *p* = 0.0017). Among infected patients, lower median Ct values were associated with higher in-hospital mortality (aOR 0.89 per Ct unit, *p* = 0.0338), while lower minimum Ct values were associated with longer hospital stay (adjusted β − 0.44 days per Ct unit, *p* = 0.0359).

**Conclusions:**

Among patients hospitalized with ADHF, concomitant SARS-CoV-2 infection was associated with substantially worse in-hospital outcomes and greater healthcare resource utilization. The observed associations between lower Ct values and adverse in-hospital outcomes should be considered hypothesis-generating and require prospective validation because Ct values are imperfect surrogate markers of viral burden.

**Supplementary Information:**

The online version contains supplementary material available at https://doi.org/10.1186/s12879-026-14407-y.

## Summary of the article’s main point

Among patients hospitalized with acute decompensated heart failure, concomitant SARS-CoV-2 infection was associated with worse in-hospital outcomes and greater resource use, while higher viral burden may further stratify mortality risk. In contrast, heart failure phenotype and pre-existing guideline-directed therapy showed no consistent associations with major in-hospitaloutcomes.

**Supplementary Information:**

The online version contains supplementary material available at https://doi.org/10.1186/s12879-026-14407-y.

## Introduction

Heart failure (HF) remains one of the leading causes of hospitalization and mortality worldwide, affecting more than 64 million individuals and imposing a substantial burden on healthcare systems [1]. Acute decompensated heart failure (ADHF) represents the most frequent cause of HF-related hospitalization and is associated with high short-term morbidity and mortality despite advances in guideline-directed medical therapy (GDMT) [2–4]. Hospitalized patients with ADHF often present with advanced age, multiple comorbidities, and impaired organ reserve, rendering them particularly vulnerable to acute systemic stressors, including infectious diseases [5, 6].

Since its emergence in late 2019, coronavirus disease 2019 (COVID-19), caused by severe acute respiratory syndrome coronavirus 2 (SARS-CoV-2), has posed unprecedented challenges to global health systems. Beyond its primary respiratory manifestations, COVID-19 has profound cardiovascular implications, including myocardial injury, arrhythmias, thromboembolic complications, and acute worsening of pre-existing cardiovascular disease [7, 8]. Patients with underlying HF represent a particularly high-risk population, with multiple studies demonstrating increased mortality and adverse outcomes in HF patients hospitalized with COVID-19 compared with non-HF populations [9–11]. Potential mechanisms underlying this vulnerability include systemic inflammation, endothelial dysfunction, myocardial injury, and increased metabolic and hemodynamic stress that may precipitate or exacerbate cardiac decompensation [12, 13].

Despite these observations, important knowledge gaps remain regarding the interaction between SARS-CoV-2 infection and acute HF syndromes. Most early studies focused on general cardiovascular cohorts or patients with chronic HF, while relatively few investigations have specifically examined patients hospitalized with ADHF. Moreover, the clinical course of COVID-19 has evolved over time due to viral mutations, changes in therapeutic strategies, and population-level immunity, raising the possibility that outcomes may vary across viral variants [14, 15]. In addition, the role of viral burden—often approximated by reverse transcription quantitative polymerase chain reaction (RT-qPCR) cycle threshold (Ct) values—as a potential determinant of disease severity and outcomes in cardiovascular patients remains incompletely understood [16].

Another unresolved question concerns the impact of pre-existing GDMT for HF on outcomes during SARS-CoV-2 infection. While HF pharmacotherapies such as β-blockers, renin–angiotensin system inhibitors, mineralocorticoid receptor antagonists, and sodium–glucose cotransporter-2 inhibitors have demonstrated substantial benefits in chronic HF populations, their influence on outcomes in patients hospitalized with ADHF and concomitant COVID-19 has not been fully clarified [2, 4, 17–19].

Therefore, the present study aimed to evaluate the clinical impact of SARS-CoV-2 infection in a large real-world cohort of patients hospitalized with ADHF at a tertiary academic center. The primary objective was to assess the association between SARS-CoV-2 infection and in-hospital mortality. Secondary objectives included ICU admission, invasive mechanical ventilation, and resource utilization. Additional exploratory analyses examined associations with Ct values, SARS-CoV-2 variant periods, HF phenotype, and pre-existing HF GDMT.

## Methods

### Study design and cohort identification

This retrospective cohort study was conducted at Knappschaft Kliniken – University Hospital Bochum, Germany, a tertiary academic center. Electronic health records were screened to identify adult patients hospitalized between 31 March 2020 and 31 December 2024 with a diagnosis of ADHF, irrespective of admission pathway (elective admission or emergency department presentation).

Eligible patients were required to be ≥ 18 years of age and to have a diagnosis of ADHF documented by ICD coding and confirmed through clinical records. ADHF was defined as an acute worsening of HF requiring escalation of HF–specific therapy, including initiation or intensification of intravenous diuretics, vasodilators, or inotropes, and—when clinically indicated—the use of mechanical circulatory or respiratory support, in accordance with contemporary guideline definitions (e.g., ESC Heart Failure Guidelines 2021 [4]).

SARS-CoV-2 infection status during the index hospitalization had to be available and was determined by reverse transcription quantitative polymerase chain reaction (RT-qPCR) testing of respiratory specimens. Infection was defined as at least one positive RT-qPCR result obtained during the same hospital stay. All included patients had clinically confirmed ADHF. SARS-CoV-2 infection was considered a concomitant exposure during the index hospitalization and was not required to be the primary reason for admission. The retrospective data did not allow a reliable distinction between patients admitted primarily because of COVID-19 and those in whom SARS-CoV-2 infection represented a concomitant or subsequently detected diagnosis. Patients without documented RT-qPCR testing were excluded to ensure a clearly defined infection status. In addition, patients were excluded when the primary reason for hospitalization was an advanced terminal condition unrelated to ADHF or COVID-19, specifically advanced malignancy or end-stage organ failure, as documented in the clinical records. This restriction was applied to avoid including hospitalizations in which short-term prognosis was predominantly determined by an unrelated terminal condition rather than ADHF or concomitant SARS-CoV-2 infection.

SARS-CoV-2 RT-qPCR testing was performed during the index hospitalization as part of routine clinical care. Testing was indicated in patients with clinical suspicion of COVID-19, including respiratory symptoms, fever, or suggestive imaging findings, and during several phases of the pandemic also as part of institutional screening protocols. Testing was not restricted to a single predefined time point. Repeated testing during hospitalization was common, particularly in cases of prolonged hospital stay or evolving clinical suspicion. The median number of RT-qPCR tests per patient was 4 (interquartile range [IQR] 2–7).

Of 3,065 initially screened hospitalizations with ADHF, a total of 2,002 patients fulfilled all eligibility criteria and were included in the final study cohort.

### Data collection and study groups

Clinical and diagnostic variables were obtained from the institutional electronic health record system. Collected data included demographic characteristics, cardiovascular risk factors and comorbidities, documented infectious complications and thromboembolic events during the index hospitalization, laboratory parameters, echocardiographic findings, baseline HF severity at admission (New York Heart Association [NYHA] functional class), as well as chronic outpatient HF therapy documented at the time of hospital admission. The diagnosis of ADHF and the corresponding clinical status were verified through detailed review of physician documentation, laboratory results (including natriuretic peptides such as NT-proBNP), imaging reports, and discharge summaries to ensure diagnostic validity. For patients who died during the index hospitalization, documented causes of death were abstracted from the available clinical records and grouped into mutually exclusive clinically relevant categories.

COVID-19 vaccination status before the index admission and the number of recorded vaccine doses were available for a subset of patients. Vaccinated status was defined as receipt of at least one documented COVID-19 vaccine dose before admission. Among SARS-CoV-2–positive patients, treatment with remdesivir during the index hospitalization was assessed from the available medication records.

Laboratory parameters were analyzed using peak values recorded during hospitalization, as these values best reflect the maximal biological disturbance and the severity of the acute decompensation.

Patients were stratified according to SARS-CoV-2 infection status during the index hospitalization into two groups: SARS-CoV-2–positive and SARS-CoV-2–negative patients. In patients with confirmed infection, additional virological parameters were evaluated. Ct values obtained from RT-qPCR testing were available for 225 of the 246 SARS-CoV-2–positive patients. Both the lowest Ct value and the median Ct value across repeated measurements during hospitalization were analyzed to approximate peak viral load and overall viral burden, respectively.

SARS-CoV-2 variant categories were assigned according to the dominant circulating variant during the respective calendar period; systematic patient-level sequencing was not available. Cases for which a variant category could not be assigned with sufficient confidence were classified as unknown.

HF phenotypes were categorized as HFrEF (LVEF ≤ 40%), HFmrEF (LVEF 41–49%), and HFpEF (LVEF ≥ 50%).

### Outcomes and exploratory analyses

The primary outcome was all-cause in-hospital mortality. Secondary clinical outcomes were ICU admission and invasive mechanical ventilation. Hospital and ICU length of stay were assessed as resource-utilization outcomes.

In patients with confirmed SARS-CoV-2 infection, the association between viral burden and clinical outcomes was explored using RT-qPCR Ct values as a surrogate marker of viral load.

Further analyses assessed in-hospital outcomes according to documented pre-admission COVID-19 vaccination status and remdesivir use among patients with available data.

Additional exploratory subgroup analyses were performed among SARS-CoV-2–positive patients to assess potential differences in clinical outcomes according to circulating viral variants and HF phenotype. Furthermore, exploratory analyses examined the association between pre-existing GDMT for HF, defined as therapy documented for ≥ 6 months prior to admission, and clinical outcomes in SARS-CoV-2–positive patients. GDMT was defined according to the contemporary four-pillar framework, comprising β-blockers, renin–angiotensin system inhibitors (angiotensin-converting enzyme inhibitors [ACEi], angiotensin receptor blockers [ARB], or angiotensin receptor–neprilysin inhibitors [ARNI]), mineralocorticoid receptor antagonists (MRA), and sodium–glucose cotransporter-2 inhibitors (SGLT2i) [2, 4, 19]. In addition, decongestive therapy, including loop and thiazide diuretics, was evaluated. Pre-admission HF medication exposure was ascertained retrospectively from medication lists and clinical documentation available at hospital admission. GDMT scores were constructed based on the number of drug classes present and analyzed as ordinal variables (range 0–4, 0–5, and 0–6). Given the limited number of patients in some subgroups, these analyses were considered exploratory and hypothesis-generating.

### Statistical analysis

Data were extracted from the hospital information system and transferred to Microsoft Excel (Microsoft Corp., Redmond, WA, USA) for preprocessing and data cleaning. Statistical analyses were performed using GraphPad Prism (version 11.0; GraphPad Software, San Diego, CA, USA) and Python (version 3.11; Python Software Foundation, Wilmington, DE, USA).

The distribution of continuous variables was assessed using the D’Agostino–Pearson omnibus normality test. Normally distributed variables are presented as mean ± standard deviation (SD), whereas non-normally distributed variables are reported as median with interquartile range (IQR). Categorical variables are summarized as counts and percentages.

Between-group comparisons were conducted using Welch’s t-test for normally distributed variables and the Mann–Whitney U test for non-normally distributed variables. For comparisons involving more than two groups, one-way analysis of variance (ANOVA) or the Kruskal–Wallis test was applied, as appropriate. Categorical variables were compared using the χ² test or Fisher’s exact test when expected cell counts were small.

Associations of SARS-CoV-2 infection status, viral burden, and pre-existing GDMT for HF with clinical outcomes were evaluated using regression analyses. Logistic regression models were applied for binary outcomes (ICU admission, invasive mechanical ventilation, and in-hospital mortality), whereas linear regression models were used for continuous outcomes (total length of hospital stay and ICU length of stay). Effect estimates are reported as odds ratios (ORs) or regression coefficients (β) with corresponding 95% confidence intervals (CIs).

Multivariable models were adjusted for clinically relevant covariates selected a priori based on biological plausibility and their potential to confound the association between SARS-CoV-2 infection and the respective outcomes. These included age, sex, diabetes mellitus, chronic kidney disease, chronic obstructive pulmonary disease, coronary artery disease, moderate-to-severe valvular heart disease, atrial fibrillation/flutter, left ventricular ejection fraction, and baseline HF severity at admission (NYHA class IV vs. III). Due to the limited number of events for invasive mechanical ventilation, a more parsimonious model including age, sex, chronic kidney disease, chronic obstructive pulmonary disease, and NYHA class (IV vs. III) was used to reduce the risk of model overfitting. Variables measured during hospitalization that could represent downstream manifestations of ADHF, SARS-CoV-2 infection, or their interaction were not included as covariates in the primary models to avoid potential overadjustment. For variables with missing data, descriptive analyses were based on available observations. Multivariable regression models were based on complete cases for the variables included in the respective model; missing values were not imputed. Multicollinearity among covariates was assessed using variance inflation factors (VIFs). Model fit and underlying assumptions were assessed using standard diagnostic procedures appropriate for logistic and linear regression models. No separate sensitivity analyses were performed.

All statistical tests were two-sided, and a p-value < 0.05 was considered statistically significant.

## Results

### Baseline characteristics

The cohort comprised 2,002 patients hospitalized with ADHF, including 246 patients (12.3%) with confirmed SARS-CoV-2 infection.

The mean age of the overall population was 74.9 ± 13.0 years and did not differ between groups. Male sex was more frequent among SARS-CoV-2–positive patients (58.9% vs. 51.7%, *p* = 0.0320). The prevalence of major cardiovascular risk factors—including diabetes mellitus, arterial hypertension, hyperlipidemia, smoking status, and obesity—was largely comparable between groups.

Similarly, the burden of comorbidities, including chronic kidney disease, chronic obstructive pulmonary disease, coronary artery disease, atrial fibrillation/flutter, moderate-to-severe valvular heart disease, and malignancy did not differ significantly between SARS-CoV-2–positive and SARS-CoV-2–negative patients. HF phenotype distribution was comparable, with HFpEF representing the predominant phenotype (67.7%), followed by HFmrEF (16.9%) and HFrEF (15.4%). Echocardiographic parameters—including left ventricular ejection fraction, right ventricular function (TAPSE), systolic pulmonary artery pressure, and the prevalence of right ventricular enlargement—were also similar across groups. Baseline functional status at admission showed a trend toward more severe symptoms among infected patients, with a higher proportion presenting in NYHA class IV (38.6% vs. 32.7%), although this difference did not reach statistical significance (*p* = 0.0648).

In contrast, several laboratory markers reflecting systemic inflammation, myocardial injury, and end-organ dysfunction were significantly higher in patients with SARS-CoV-2 infection. These included peak C-reactive protein, leukocyte count, NT-proBNP, troponin, creatine kinase, and lactate dehydrogenase (all *p* < 0.01). In addition, markers of renal and hepatic dysfunction were more pronounced among infected patients, as reflected by higher peak creatinine levels, lower nadir eGFR, and elevated liver enzymes and cholestatic parameters, indicating greater multiorgan involvement in the context of SARS-CoV-2 infection.

Chronic HF pharmacotherapy at admission was broadly similar between groups, including the use of β-blockers, renin–angiotensin system inhibitors, mineralocorticoid receptor antagonists, and loop diuretics. However, sodium–glucose cotransporter-2 inhibitor therapy—despite its overall low utilization—was more frequent among SARS-CoV-2–positive patients (13.4% vs. 7.2%, *p* = 0.0022). The overall burden of GDMT did not differ significantly between groups.

COVID-19 vaccination status before the index admission was available for 681 of 2,002 patients (34.0%), including 59 of 246 SARS-CoV-2–positive patients (24.0%) and 622 of 1,756 SARS-CoV-2–negative patients (35.4%). Among patients with available data, 635 (93.2%) had received at least one documented vaccine dose before admission; vaccination uptake was 89.8% among SARS-CoV-2–positive and 93.6% among SARS-CoV-2–negative patients (*p* = 0.2748). Among vaccinated patients, 28 (4.4%) had received one recorded dose and 607 (95.6%) two recorded doses.

Among infected patients, SARS-CoV-2 viral load parameters during hospitalization showed a median Ct value of 29.5 (IQR 26.0–33.0) and a lowest Ct value of 22.5 (IQR 19.3–30.5), indicating substantial interindividual variability in viral burden. A detailed overview of baseline characteristics is provided in Table 1.

**Table 1:** Baseline characteristics of patients hospitalized with acute decompensated heart failure according to SARS-CoV-2 infection status

Variable	Total (*n* = 2002)	SARS-CoV-2 positive (*n* = 246)	SARS-CoV-2 negative (*n* = 1756)	*p*-value
**Demographics**				
Age (years)	74.9 ± 13.0	74.9 ± 12.7	74.9 ± 13.0	0.9640
Male sex	1052 (52.5)	145 (58.9)	907 (51.7)	**0.0320**
***Cardiovascular Risk Factors***				
Diabetes mellitus	674 (33.7)	95 (38.6)	579 (33.0)	0.0793
Hypertension	711 (35.5)	90 (36.6)	621 (35.4)	0.7078
Smoking	106 (5.3)	7 (2.8)	99 (5.6)	0.0670
Hyperlipidemia	576 (28.8)	63 (25.6)	513 (29.2)	0.2422
Obesity	211 (10.5)	22 (8.9)	189 (10.8)	0.3839
BMI (kg/m²)	28.2 ± 6.4 (*N* = 880)	28.0 ± 5.2 (*N* = 71)	28.2 ± 6.5 (*N* = 809)	0.8268
***Comorbidities***				
CKD	421 (21.0)	50 (20.3)	371 (21.1)	0.7724
COPD	310 (15.5)	31 (12.6)	279 (15.9)	0.1820
CAD	880 (44.0)	104 (42.3)	776 (44.2)	0.5709
Moderate-to-severe valvular heart disease	463 (23.1)	57 (23.2)	406 (23.1)	0.9861
Atrial fibrillation/flutter	951 (47.5)	116 (47.2)	835 (47.6)	0.9071
Malignancy	187 (9.3)	15 (6.1)	172 (9.8)	0.0620
***Heart Failure Characteristics***				
HFrEF	207 (15.4) (*N* = 1344)	19 (14.3) (*N* = 133)	188 (15.5) (*N* = 1211)	0.7072
HFmrEF	227 (16.9) (*N* = 1344)	23 (17.3) (*N* = 133)	204 (16.9) (*N* = 1211)	0.8959
HFpEF	910 (67.7) (*N* = 1344)	91 (68.4) (*N* = 133)	819 (67.6) (*N* = 1211)	0.8531
NYHA class III at admission	1333 (66.6)	151 (61.4)	1182 (67.3)	0.0648
NYHA class IV at admission	669 (33.4)	95 (38.6)	574 (32.7)	0.0648
LVEF (%)	55.0 (45.0–55.0) (*N* = 1344)	55.0 (45.0–55.0) (*N* = 133)	55.0 (45.0–55.0) (*N* = 1211)	0.9165
TAPSE (mm)	17.0 (17.0–18.0) (*N* = 1309)	17.0 (17.0–18.0) (*N* = 132)	17.0 (17.0–18.0) (*N* = 1177)	0.9649
sPAP (mmHg)	30.0 (24.0–31.0) (*N* = 1025)	30.0 (26.0–30.0) (*N* = 111)	30.0 (24.0–32.0) (*N* = 914)	0.2242
Right ventricular enlargement	236 (18.1) (*N* = 1301)	20 (14.8) (*N* = 135)	216 (18.5) (*N* = 1166)	0.2896
***Laboratory Parameters (Peak Values During Hospitalization)***				
Peak creatinine (mg/dL)	1.6 (1.2–2.5)	1.9 (1.3–2.7)	1.6 (1.2–2.5)	**0.0004**
Nadir eGFR (mL/min/1.73 m²)	37.1 (23.0–57.0)	32.5 (21.2–50.3)	37.9 (23.2–58.5)	**0.0033**
Peak CRP (mg/dL)	9.7 (2.0–22.6)	17.6 (5.9–29.2)	8.7 (1.7–21.3)	**< 0.0001**
Peak leukocytes (10^9/L)	12.8 (8.8–19.0)	15.2 (10.0–22.5)	12.4 (8.7–18.5)	**< 0.0001**
Peak procalcitonin (ng/mL)	1.0 (0.2–6.6) (*N* = 1298)	1.3 (0.3–7.0) (*N* = 201)	0.9 (0.2–6.3) (*N* = 1097)	0.1272
Peak NT-proBNP (pg/mL)	4502.0 (1641.0–13060.0)	5204.0 (2400.0–17560.0)	4333.0 (1531.0–12603.0)	**0.0016**
Peak troponin (ng/L)	54.0 (27.1–143.2) (*N* = 1401)	72.2 (33.3–190.4) (*N* = 173)	52.0 (26.3–138.2) (*N* = 1228)	**0.0061**
Peak CK (U/L)	152.0 (71.6–474.2)	226.3 (84.0–730.3)	143.8 (70.5– 426.9)	**< 0.0001**
Peak CK-MB (U/L)	33.4 (20.7–69.7)	38.1 (21.1–74.5)	32.2 (20.7–68.6)	0.2908
Peak ALT (U/L)	34.2 (19.0–85.7)	53.2 (26.9–132.5)	31.9 (18.2–78.1)	**< 0.0001**
Peak AST (U/L)	50.6 (26.7–148.1)	89.8 (39.6–268.7)	46.9 (25.9–130.6)	**< 0.0001**
Peak bilirubin (mg/dL)	0.8 (0.5–1.5)	1.0 (0.7–2.0)	0.8 (0.5–1.4)	**< 0.0001**
Peak LDH (U/L)	322.0 (244.0–498.0)	411.0 (279.5–764.5)	315.0 (240.8–469.0)	**< 0.0001**
Peak GGT (U/L)	87.5 (38.0– 207.3)	141.0 (58.0–287.0)	81.0 (36.0– 197.0)	**< 0.0001**
Peak ALP (U/L)	117.0 (85.0–181.0)	144.0 (92.0–226.0)	114.0 (84.0– 173.0)	**< 0.0001**
Peak GLDH (U/L)	14.3 (4.5–54.5) (*N* = 929)	20.1 (6.7–74.6) (*N* = 130)	13.4 (4.3–50.9) (*N* = 799)	**0.0111**
***SARS-CoV-2 Viral Load Parameters during hospitalization***				
Lowest SARS-CoV-2 PCR Ct value		22.5 (19.3–30.5) (*N* = 225)		
Median SARS-CoV-2 PCR Ct value		29.5 (26.0–33.0) (*N* = 225)		
***Heart Failure Medication at Admission***				
Beta-blocker use	1057 (60.9) (*N* = 1737)	129 (64.2) (*N* = 201)	928 (60.4) (*N* = 1536)	0.3041
ACEi/ARB/ARNI use	861 (49.6) (*N* = 1737)	94 (46.8) (*N* = 201)	767 (49.9) (*N* = 1536)	0.3981
MRA use	309 (17.8) (*N* = 1737)	37 (18.4) (*N* = 201)	272 (17.7) (*N* = 1536)	0.8073
SGLT2i use	138 (7.9) (*N* = 1737)	27 (13.4) (*N* = 201)	111 (7.2) (*N* = 1536)	**0.0022**
Loop diuretic use	892 (51.4) (*N* = 1737)	107 (53.2) (*N* = 201)	785 (51.1) (*N* = 1536)	0.5705
Thiazide diuretic use	134 (7.7) (*N* = 1737)	11 (5.5) (*N* = 201)	123 (8.0) (*N* = 1536)	0.2053
GDMT-related drug classes*	2.0 (1.0–3.0) (*N* = 1737)	2.0 (1.0–3.0) (*N* = 201)	2.0 (1.0–3.0) (*N* = 1536)	0.3806
***COVID-19 vaccination***				
Vaccinated before index admission	635 (93.2) (*N* = 681)	53 (89.8) (*N* = 59)	582 (93.6) (*N* = 622)	0.2748
One recorded vaccine dose	28 (4.4) (*N* = 635)	1 (1.9) (*N* = 53)	27 (4.6) (*N* = 582)	0.5011
Two recorded vaccine doses	607 (95.6) (*N* = 635)	52 (98.1) (*N* = 53)	555 (95.4) (*N* = 582)	0.5011

*GDMT indicates guideline-directed medical therapy. The score represents the number of HF drug classes present in chronic outpatient medication prior to hospital admission and was calculated as the sum of β-blocker, ACEi/ARB/ARNI, MRA, SGLT2i, and loop diuretic therapy (1 = present, 0 = absent; range 0–5)

Data are presented as mean ± standard deviation, median (interquartile range), or n (%), as appropriate. Continuous variables were compared using Welch’s t-test or Mann–Whitney U test; categorical variables were analyzed using the χ² test or Fisher’s exact test, as appropriate. Peak laboratory values represent the highest recorded value during hospitalization. SARS-CoV-2 viral load parameters were assessed only in infected patients. For variables with incomplete data, the number of available observations is indicated, and analyses were performed using available-case data

Abbreviations: ACEi, angiotensin-converting enzyme inhibitor; ALP, alkaline phosphatase; ALT, alanine aminotransferase; ARB, angiotensin receptor blocker; ARNI, angiotensin receptor–neprilysin inhibitor; AST, aspartate aminotransferase; BMI, body mass index; CAD, coronary artery disease; CK, creatine kinase; CKD, chronic kidney disease; CK-MB, creatine kinase–myocardial band; COPD, chronic obstructive pulmonary disease; CRP, C-reactive protein; Ct, cycle threshold; eGFR, estimated glomerular filtration rate; GDMT, guideline-directed medical therapy; GGT, gamma-glutamyl transferase; GLDH, glutamate dehydrogenase; HFmrEF, heart failure with mildly reduced ejection fraction; HFpEF, heart failure with preserved ejection fraction; HFrEF, heart failure with reduced ejection fraction; LDH, lactate dehydrogenase; LVEF, left ventricular ejection fraction; MRA, mineralocorticoid receptor antagonist; NT-proBNP, N-terminal pro–B-type natriuretic peptide; NYHA, New York Heart Association; SARS-CoV-2, severe acute respiratory syndrome coronavirus 2; SGLT2i, sodium–glucose cotransporter 2 inhibitor; sPAP, systolic pulmonary artery pressure; TAPSE, tricuspid annular plane systolic excursion

### Association of SARS-CoV-2 infection with in-hospital outcomes

Patients with concomitant SARS-CoV-2 infection experienced significantly worse in-hospital outcomes than SARS-CoV-2–negative patients (Table 2). In-hospital mortality was significantly higher in SARS-CoV-2–positive than in SARS-CoV-2–negative patients (32.5% vs. 20.6%, *p* < 0.0001). Among SARS-CoV-2–positive decedents, pneumonia (42.5%) and heart failure (30.0%) were the most frequent documented causes of death and occurred more frequently than among SARS-CoV-2–negative decedents (22.2%, *p* = 0.0002, and 19.9%, *p* = 0.0486, respectively). Detailed results are provided in Supplementary Table 1.

**Table 2:** In-hospital outcomes of patients hospitalized with acute decompensated heart failure according to SARS-CoV-2 infection status

Variable	Total (*n* = 2002)	SARS-CoV-2 positive (*n* = 246)	SARS-CoV-2 negative (*n* = 1756)	*p*-value
ICU admission	1042 (52.0)	151 (61.4)	891 (50.7)	**0.0018**
Invasive mechanical ventilation	58 (2.9)	16 (6.5)	42 (2.4)	**0.0003**
In-hospital mortality	441 (22.0)	80 (32.5)	361 (20.6)	**< 0.0001**
Length of hospital stay (days)	13.0 (8.0–23.0)	21.5 (12.0–33.3)	12.0 (7.0–21.0)	**< 0.0001**
ICU length of stay (days)	14.0 (7.0–25.0)	22.0 (10.0–35.0)	13.0 (6.0–23.0)	**< 0.0001**

Data are presented as n (%) or median (interquartile range), as appropriate. Categorical variables were compared using the χ² test, and continuous variables using the Mann–Whitney U test

Abbreviations: ICU, intensive care unit; SARS-CoV-2, severe acute respiratory syndrome coronavirus 2

ICU admission (61.4% vs. 50.7%, *p* = 0.0018) and invasive mechanical ventilation (6.5% vs. 2.4%, *p* = 0.0003) were also more frequent among infected patients. Resource utilization was also greater among SARS-CoV-2–positive patients, with longer hospital stays (21.5 [12.0–33.3] vs. 12.0 [7.0–21.0] days, *p* < 0.0001) and ICU stays (22.0 [10.0–35.0] vs. 13.0 [6.0–23.0] days, *p* < 0.0001).

**Fig. 1:**
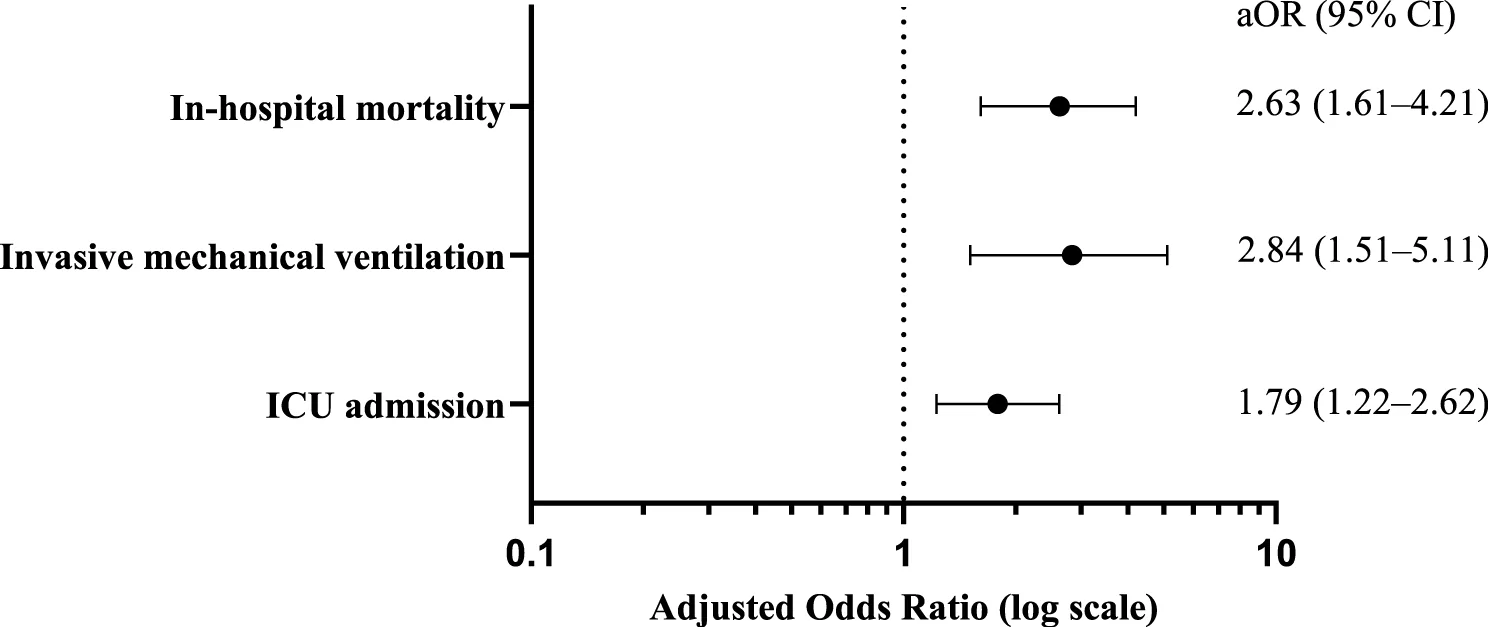
Adjusted associations between SARS-CoV-2 infection and in-hospital outcomes in patients hospitalized with acute decompensated heart failure. Forest plot showing adjusted odds ratios (aORs) with 95% confidence intervals (CI) for ICU admission, invasive mechanical ventilation, and in-hospital mortality associated with SARS-CoV-2 infection. Multivariable logistic regression models were adjusted for age, sex, diabetes mellitus, chronic kidney disease, chronic obstructive pulmonary disease, coronary artery disease, moderate-to-severe valvular heart disease, atrial fibrillation/flutter, left ventricular ejection fraction, and NYHA class IV at admission (vs. NYHA III). Due to limited number of events, invasive mechanical ventilation was analyzed using a parsimonious model adjusted for age, sex, chronic kidney disease, chronic obstructive pulmonary disease, and NYHA class IV at admission (vs. NYHA class III). The vertical dashed line indicates OR of 1.0 (no association). Abbreviations: ICU, intensive care unit; NYHA, New York Heart Association; aOR, adjusted odds ratio; CI, confidence interval; SARS-CoV-2, severe acute respiratory syndrome coronavirus 2

**Fig. 2:**
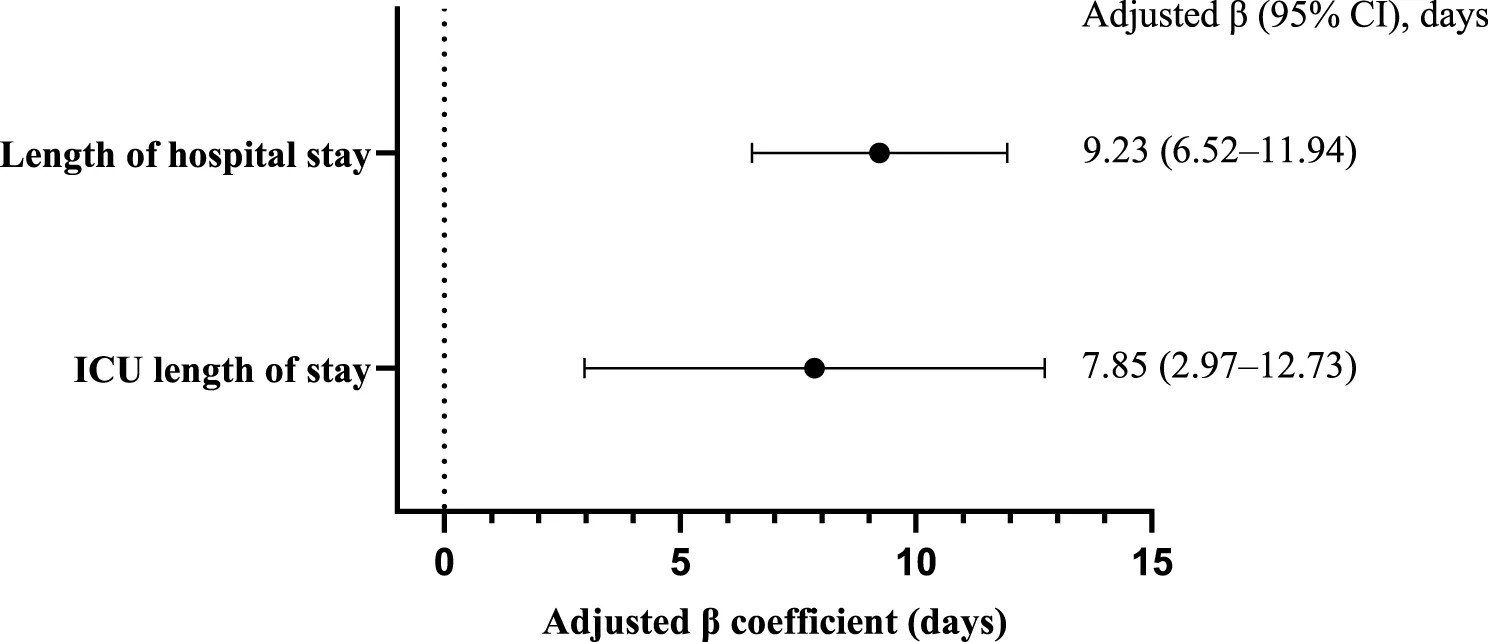
Adjusted association between SARS-CoV-2 infection and resource-utilization outcomes in patients hospitalized with acute decompensated heart failure. Forest plot showing adjusted β coefficients (days) with 95% confidence intervals (CI) for the association between SARS-CoV-2 infection and length of hospital stay as well as ICU length of stay as measures of healthcare resource utilization. Estimates were derived from multivariable linear regression models adjusted for age, sex, diabetes mellitus, chronic kidney disease, chronic obstructive pulmonary disease, coronary artery disease, moderate-to-severe valvular heart disease, atrial fibrillation/flutter, left ventricular ejection fraction, and NYHA class IV at admission (vs. NYHA class III). The vertical dashed line indicates no association (β = 0). Abbreviations: ICU, intensive care unit; NYHA, New York Heart Association; CI, confidence interval; SARS-CoV-2, severe acute respiratory syndrome coronavirus 2

In regression analyses, SARS-CoV-2 infection was consistently associated with adverse in-hospital outcomes. After multivariable adjustment, SARS-CoV-2 infection remained associated with in-hospital mortality (adjusted OR 2.63, 95% CI 1.61–4.21; *p* = 0.0002), ICU admission (adjusted OR 1.79, 95% CI 1.22–2.62; *p* = 0.0027), and invasive mechanical ventilation (adjusted OR 2.84, 95% CI 1.51–5.11; *p* = 0.0017) (Fig. 1). SARS-CoV-2 infection was also associated with greater resource utilization, reflected by longer hospital stay (adjusted β 9.23 days, 95% CI 6.52–11.94; *p* < 0.0001) and ICU stay (adjusted β 7.85 days, 95% CI 2.97–12.73; *p* = 0.0017) (Fig. 2). Full regression results are provided in Supplementary Table 2.

In exploratory analyses restricted to patients with available vaccination data, in-hospital outcomes did not differ significantly according to documented pre-admission COVID-19 vaccination status (all *p* > 0.10). Among SARS-CoV-2–positive patients, 28 of 246 (11.4%) received remdesivir during the index hospitalization. In exploratory analyses, remdesivir use was not significantly associated with in-hospital mortality, ICU admission, invasive mechanical ventilation, or resource-utilization outcomes (all *p* > 0.10).

### Infectious complications and thromboembolic events during hospitalization

Rates of bacterial pneumonia (25.6% vs. 22.9%, *p* = 0.3446), urinary tract infection (6.1% vs. 3.8%, *p* = 0.0812), bloodstream infection/bacteremia (8.1% vs. 5.2%, *p* = 0.0646), and sepsis (23.6% vs. 18.7%, *p* = 0.0717) did not differ significantly between SARS-CoV-2–positive and SARS-CoV-2–negative patients. Other documented respiratory viral infections were rare and occurred exclusively in SARS-CoV-2–negative patients, comprising one influenza virus infection, one adenovirus infection, and three human metapneumovirus infections; no RSV (respiratory syncytial virus) or parainfluenza virus infections were documented. Consistent with these findings, peak procalcitonin levels did not differ significantly between groups (1.3 [0.3–7.0] vs. 0.9 [0.2–6.3] ng/mL, *p* = 0.1272). Documented infectious complications during the index hospitalization are summarized in Supplementary Table 3. Thromboembolic events were uncommon and did not differ significantly between SARS-CoV-2–positive and SARS-CoV-2–negative patients (2.0% vs. 3.1%, *p* = 0.3651). The distribution of individual thromboembolic events is provided in Supplementary Table 4.

### Association of SARS-CoV-2 viral burden with clinical outcomes

**Fig. 3:**
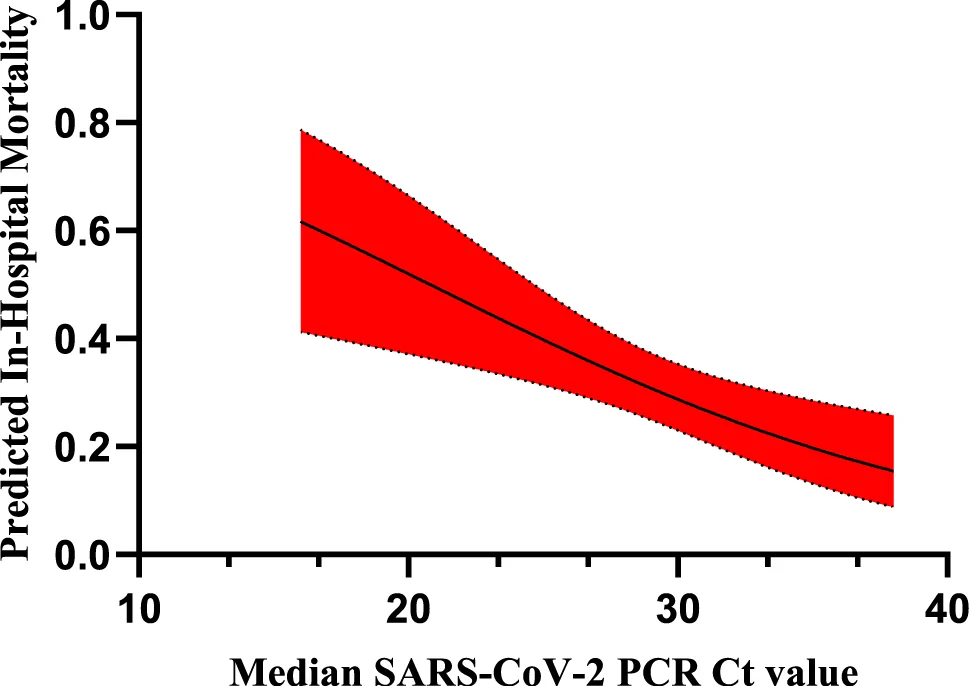
Adjusted relationship between median SARS-CoV-2 PCR Ct values and predicted in-hospital mortality in patients with acute decompensated heart failure. The black line represents model-estimated mortality risk, and the shaded area indicates the 95% confidence interval. Estimates were derived from multivariable logistic regression model adjusted for age, sex, diabetes mellitus, chronic kidney disease, chronic obstructive pulmonary disease, coronary artery disease, moderate-to-severe valvular heart disease, atrial fibrillation/flutter, left ventricular ejection fraction, and NYHA class IV at admission (vs. NYHA class III). Lower Ct values indicate higher viral load. Abbreviations: Ct, cycle threshold; NYHA, New York Heart Association; SARS-CoV-2, severe acute respiratory syndrome coronavirus 2

Among SARS-CoV-2–positive patients, median Ct values during hospitalization were significantly lower in non-survivors than in survivors (28.0 [23.3–32.4] vs. 30.5 [27.0–33.4], *p* = 0.0041), indicating a higher persistent viral burden in patients who died during hospitalization (Supplementary Fig. 1). Consistently, in regression analyses, higher median Ct values were associated with lower odds of in-hospital mortality, both in univariable analysis (OR 0.91, 95% CI 0.85–0.96; *p* = 0.0012) and after multivariable adjustment (adjusted OR 0.89, 95% CI 0.79–0.99; *p* = 0.0338) (Fig. 3).

**Fig. 4:**
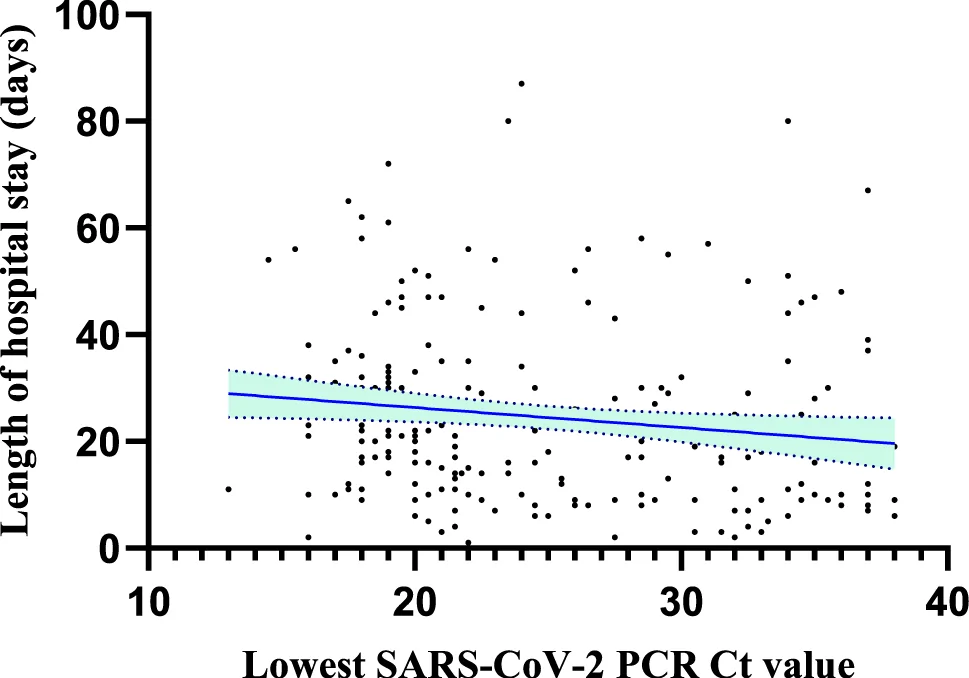
Adjusted association between lowest SARS-CoV-2 PCR Ct values and length of hospital stay in patients with acute decompensated heart failure. Scatter plot illustrating the relationship between the lowest SARS-CoV-2 PCR Ct value during hospitalization and length of hospital stay. The solid line represents the adjusted regression estimate, and the shaded area indicates the 95% confidence interval. Estimates were derived from multivariable linear regression model adjusted for age, sex, diabetes mellitus, chronic kidney disease, chronic obstructive pulmonary disease, coronary artery disease, moderate-to-severe valvular heart disease, atrial fibrillation/flutter, left ventricular ejection fraction, and NYHA class IV at admission (vs. NYHA class III). Lower Ct values indicate higher viral load. Abbreviations: Ct, cycle threshold; NYHA, New York Heart Association; SARS-CoV-2, severe acute respiratory syndrome coronavirus 2

In addition, the lowest Ct value recorded during hospitalization was independently associated with length of hospital stay. Higher lowest Ct values, reflecting lower peak viral load, were associated with shorter hospitalization in both univariable analysis (β − 0.37 days, 95% CI − 0.70 to − 0.05; *p* = 0.0250) and after multivariable adjustment (adjusted β − 0.44 days, 95% CI − 0.84 to − 0.03; *p* = 0.0359) (Fig. 4). SARS-CoV-2 viral load parameters were not significantly associated with the remaining primary or secondary outcomes.

Together, these findings suggest that higher persistent and peak SARS-CoV-2 viral burden may be associated with adverse clinical trajectories in patients hospitalized with ADHF and concomitant SARS-CoV-2 infection.

### Variant-specific differences in in-hospital outcomes among patients with ADHF and SARS-CoV-2 infection

Among patients with ADHF and confirmed SARS-CoV-2 infection, in-hospital outcomes differed significantly according to viral variant (Table 3; Supplementary Fig. 2). Patients infected during the wild-type, Alpha, and Delta phases showed the highest rates of ICU admission, invasive mechanical ventilation, and in-hospital mortality, whereas outcomes were generally more favorable during the Omicron era, particularly for BA.1 and BA.2. ICU admission ranged from 56.3% to 91.7% across known variant groups (*p* = 0.0036), invasive mechanical ventilation from 0.0% to 20.8% (*p* = 0.0241), and in-hospital mortality from 14.3% to 58.3% (*p* = 0.0345).

**Table 3:** Variant-specific in-hospital outcomes among patients with acute decompensated heart failure and SARS-CoV-2 infection

Variable	Total SARS-CoV-2 positive (*n* = 246)	Wild-type (*n* = 24)	Alpha (*n* = 10)	Delta (*n* = 12)	Omicron BA.1 (*n* = 32)	Omicron BA.2 (*n* = 14)	Omicron BA.5 (*n* = 81)	Unknown variant (*n* = 73)	*p*-value
ICU admission	151 (61.4)	22 (91.7)	9 (90.0)	11 (91.7)	18 (56.3)	9 (64.3)	48 (59.3)	34 (46.6)	**0.0036**
Invasive mechanical ventilation	16 (6.5)	5 (20.8)	2 (20.0)	2 (16.7)	4 (12.5)	0 (0.0)	3 (3.7)	0 (0.0)	**0.0241**
In-hospital mortality	80 (32.5)	12 (50.0)	4 (40.0)	7 (58.3)	6 (18.8)	2 (14.3)	27 (33.3)	22 (30.1)	**0.0345**
Length of hospital stay (days)	21.5 (12.0– 33.3)	36.5 (18.8–41.8)	38.0 (13.5–57.3)	24.0 (13.3–49.3)	30.0 (17.8–43.5)	23.5 (13.5–44.0)	20.0 (10.0–30.0)	17.0 (9.0– 26.5)	**0.0148**
ICU length of stay (days)	22.0 (10.0– 35.0)	30.0 (15.5–41.3)	45.0 (16.5–57.0)	23.0 (12.0–43.0)	33.5 (20.3–50.3)	22.0 (11.5–47.5)	17.0 (8.3–26.8)	13.0 (7.8– 27.3)	**0.0047**

Data are presented as n (%) or median (interquartile range), as appropriate. Continuous variables were compared using Kruskal–Wallis test, and categorical variables using Fisher’s exact test. Statistical comparisons were performed only among patients with known SARS-CoV-2 variants; cases with unknown variant status are shown for completeness but were excluded from the statistical testing

Abbreviations: ICU, intensive care unit; SARS-CoV-2, severe acute respiratory syndrome coronavirus 2

A similar pattern was observed for resource utilization. Length of hospital stay and ICU stay varied significantly across variant groups, with the longest stays seen in earlier pandemic phases and shorter stays during later Omicron sublineages (*p* = 0.0148 and *p* = 0.0047, respectively). Given the limited number of patients in several variant-specific subgroups, these findings should be interpreted cautiously and considered primarily descriptive and hypothesis-generating.

### In-hospital outcomes according to HF phenotype in SARS-CoV-2–positive patients

Among SARS-CoV-2–positive patients with ADHF, major in-hospital outcomes were broadly comparable across HF phenotypes (Table 4; Supplementary Fig. 3). Rates of ICU admission, invasive mechanical ventilation, and in-hospital mortality did not differ significantly between patients with HFrEF, HFmrEF, and HFpEF.

**Table 4:** In-hospital outcomes among SARS-CoV-2–positive patients with acute decompensated heart failure according to heart failure phenotype

Variable	Total SARS-CoV-2 positive (*n* = 246)	HFrEF (*n* = 19)	HFmrEF (*n* = 23)	HFpEF (*n* = 91)	Unknown HF phenotype (*n* = 113)	*p*-value
ICU admission	151 (61.4)	8 (42.1)	9 (39.1)	50 (54.9)	84 (74.3)	0.3128
Invasive mechanical ventilation	16 (6.5)	0 (0.0)	0 (0.0)	4 (4.4)	12 (10.6)	0.7757
In-hospital mortality	80 (32.5)	6 (31.6)	4 (17.4)	20 (22.0)	50 (44.2)	0.5681
Length of hospital stay (days)	21.5 (12.0–33.3)	11.0 (8.0–19.0)	25.0 (17.0–26.0)	27.0 (15.0–40.0)	20.0 (11.0–32.0)	**0.0006**
ICU length of stay (days)	22.0 (10.0–35.0)	9.5 (7.0–16.3)	25.0 (20.5–38.0)	29.0 (12.8–41.3)	18.0 (8.3–32.8)	**0.0255**

Data are presented as n (%) or median (interquartile range), as appropriate. Continuous variables were compared using Kruskal–Wallis test; categorical variables using Fisher’s exact test. Statistical comparisons were performed only among patients with a known heart failure phenotype (HFrEF, HFmrEF, HFpEF); patients with unknown phenotype are shown for completeness but were excluded from statistical testing

Abbreviations: ICU, intensive care unit; SARS-CoV-2, severe acute respiratory syndrome coronavirus 2; HFrEF, heart failure with reduced ejection fraction; HFmrEF, heart failure with mildly reduced ejection fraction; HFpEF, heart failure with preserved ejection fraction

In contrast, measures of resource utilization differed across phenotypes. Median length of hospital stay varied significantly (*p* = 0.0006), with shorter hospitalization observed in patients with HFrEF and longer stays in HFmrEF and HFpEF. Similarly, ICU length of stay differed across phenotypes (*p* = 0.0255), with shorter ICU stays in HFrEF and longer stays in HFmrEF and HFpEF. Given the limited number of patients in phenotype subgroups, these findings should be interpreted cautiously and considered primarily exploratory and hypothesis-generating.

### HF GDMT and in-hospital outcomes in SARS-CoV-2–positive patients

Among SARS-CoV-2–positive patients, most chronic HF medication classes were not independently associated with major in-hospital outcomes. In regression analyses, pre-admission β-blocker therapy was associated with lower odds of ICU admission in both univariable (OR 0.32, 95% CI 0.16–0.60; *p* = 0.0003) and multivariable analyses (adjusted OR 0.27, 95% CI 0.10–0.67; *p* = 0.0043). Loop diuretic therapy was associated with a lower likelihood of invasive mechanical ventilation in univariable analysis (OR 0.18, 95% CI 0.04–0.58; *p* = 0.0031) and remained significant after multivariable adjustment (adjusted OR 0.24, 95% CI 0.05–0.89; *p* = 0.0314).

Several associations observed in univariable analyses did not persist after adjustment. In particular, the inverse association between β-blocker therapy and in-hospital mortality (OR 0.49, 95% CI 0.26–0.93; *p* = 0.0280), as well as the association between loop diuretic therapy and ICU admission (OR 0.54, 95% CI 0.30–0.95; *p* = 0.0339), were attenuated in multivariable models.

A higher GDMT score (range 0–5)—comprising β-blockers, ACEi/ARB/ARNI, MRA, SGLT2 inhibitors, and loop diuretics—was associated with lower odds of ICU admission (OR 0.74, 95% CI 0.59–0.94; *p* = 0.0110) and invasive mechanical ventilation (OR 0.59, 95% CI 0.36–0.91; *p* = 0.0166), and showed a non-significant trend toward lower in-hospital mortality (OR 0.80, 95% CI 0.62–1.02; *p* = 0.0770) in univariable analyses; however, these associations were no longer significant after multivariable adjustment. Overall, in these exploratory analyses, neither the conventional four-pillar HF therapy score nor extended GDMT scores incorporating loop diuretics or both loop and thiazide diuretics demonstrated independent associations with adverse in-hospital outcomes.

## Discussion

In this cohort of patients hospitalized with ADHF, concomitant SARS-CoV-2 infection remained associated with higher in-hospital mortality after multivariable adjustment and was also associated with the secondary clinical outcomes of ICU admission and invasive mechanical ventilation. Infected patients showed greater healthcare resource utilization, reflected by longer hospital and ICU stays. These findings extend prior evidence that HF is a major vulnerability factor in COVID-19 and suggest that, in patients admitted with ADHF, SARS-CoV-2 infection acts as an additional clinically relevant stressor rather than a coincidental comorbidity. This is biologically plausible given the limited physiological reserve of ADHF patients and the known cardiovascular effects of COVID-19, including systemic inflammation, endothelial dysfunction, myocardial injury, thromboinflammation, and multiorgan involvement [20–22].

SARS-CoV-2–positive patients showed more pronounced inflammatory, cardiac, renal, and hepatic laboratory abnormalities during hospitalization. These findings are compatible with a more severe systemic illness in patients with concomitant ADHF and COVID-19; however, they should be interpreted cautiously because these measurements represent peak or nadir values obtained during the hospital course. Accordingly, these abnormalities may reflect the combined effects of cardiac decompensation, COVID-19–related systemic illness, and subsequent organ dysfunction rather than a direct effect of SARS-CoV-2 itself. From a pathophysiological perspective, inflammatory and hemodynamic stress may exacerbate congestion, impair myocardial reserve, and promote secondary end-organ injury in patients with ADHF [12, 23, 24]; however, the present observational data do not allow the relative contribution of these mechanisms to the observed outcomes to be determined. No significant between-group differences were observed in documented bacterial infections or thromboembolic events, suggesting that these complications were unlikely to explain the observed outcome differences.

Importantly, SARS-CoV-2 infection remained associated with more than twofold higher odds of in-hospital death after multivariable adjustment, underscoring the vulnerability of patients hospitalized with ADHF. This extends previous findings from broader HF and cardiovascular cohorts to a clinically defined ADHF population [25, 26].

An additional strength of our study is the integration of virological burden into the clinical analysis. Among SARS-CoV-2–positive patients, lower median Ct values during hospitalization, reflecting higher persistent viral burden, were associated with in-hospital mortality, while lower nadir Ct values were associated with longer hospitalization. These findings suggest that sustained viral burden may identify a subgroup of ADHF patients at particularly high risk for adverse in-hospital trajectories. Prior studies and systematic reviews have reported associations between lower Ct values and greater COVID-19 severity or mortality, although the strength and consistency of these relationships vary across studies [27, 28]. Our data extend this concept to the setting of ADHF, where persistent viral burden may reflect a combination of impaired host resilience, prolonged viral replication, and greater systemic inflammatory activation. Importantly, Ct values remain an imperfect surrogate of viral burden because they depend on assay platform, specimen quality, sampling timing, and viral kinetics. Accordingly, the observed associations should be considered exploratory and hypothesis-generating and do not support the use of Ct values for clinical risk stratification or treatment decisions without prospective validation [16, 29, 30].

We observed clinically relevant differences in outcomes across SARS-CoV-2 variant phases, with worse outcomes during earlier pandemic waves and more favorable courses during Omicron-dominant periods, particularly BA.1 and BA.2. This pattern is consistent with large population-based studies demonstrating lower risks of severe disease, ICU admission, and mortality during Omicron compared with Delta and earlier variants [14, 31, 32]. However, these differences cannot be attributed to intrinsic variant virulence alone, as increasing population immunity, vaccination coverage, prior infections, evolving testing strategies, and changes in standards of care occurred concurrently [33]. Moreover, because variant assignment was based on the dominant circulating variant during predefined calendar periods rather than systematic patient-level sequencing, some degree of variant misclassification cannot be excluded. Accordingly, our variant-specific findings should be interpreted cautiously and primarily as descriptive. Nevertheless, they highlight that the interaction between ADHF and SARS-CoV-2 infection has evolved over the course of the pandemic and that temporal context is essential when interpreting outcome estimates in COVID-19–related cardiovascular research.

Major in-hospital outcomes were broadly comparable across HF phenotypes, although hospital and ICU stays differed. This suggests that short-term outcomes in patients with ADHF and concomitant SARS-CoV-2 infection may be driven more by the acute combined cardiac and infectious disease burden than by HF phenotype alone [25, 26]. This interpretation is plausible given that ADHF, irrespective of ejection fraction category, shares common pathophysiological pathways including congestion, neurohormonal activation, organ dysfunction, and heightened susceptibility to inflammatory stress [34, 35]. However, given the limited subgroup sizes, these findings should be interpreted cautiously and considered exploratory.

Our exploratory, hypothesis-generating analyses of pre-admission HF GDMT [2, 4, 19] should be interpreted cautiously. Most chronic HF medication classes and composite GDMT scores were not independently associated with major in-hospital outcomes after multivariable adjustment. These findings suggest that the overall burden of pre-existing HF pharmacotherapy may not be a dominant determinant of short-term prognosis once patients are hospitalized with ADHF and concomitant SARS-CoV-2 infection. This observation is consistent with the concept that, although GDMT substantially improves long-term outcomes in chronic HF, its protective effects may be attenuated in the setting of acute systemic illness such as severe viral infection [23, 25, 35, 36]. The observed associations of beta-blocker therapy with lower ICU admission and loop diuretic therapy with reduced risk of invasive mechanical ventilation should therefore be interpreted as exploratory and may reflect residual confounding by indication or differences in baseline HF stability. Importantly, these findings do not challenge the well-established long-term prognostic benefits of GDMT demonstrated in randomized trials and reflected in contemporary HF guidelines [2, 4, 18].

From a clinical perspective, our findings support heightened vigilance when ADHF and SARS-CoV-2 infection coexist. Patients with this dual burden represent a high-risk inpatient population with greater escalation-of-care requirements and excess mortality, supporting close respiratory and hemodynamic monitoring and timely multidisciplinary management.

### Limitations

This study has several limitations that should be considered when interpreting the findings. First, the retrospective single-center design is inherently susceptible to residual confounding despite multivariable adjustment. Second, the study period spanned multiple pandemic phases, during which testing strategies, admission thresholds, supportive care practices, and antiviral therapies evolved and may have influenced both exposure classification and clinical outcomes. Although remdesivir treatment could be assessed, detailed patient-level information on other COVID-19–specific therapies, including corticosteroids, nirmatrelvir/ritonavir, IL-6 inhibitors, and other immunomodulatory agents, was not systematically available and could therefore not be incorporated into the analyses. Third, Ct values were used as surrogate markers of viral burden; however, they are assay- and sampling-dependent and may be influenced by specimen quality, timing of collection, viral kinetics, laboratory platform, and repeated testing practices. Ct values were also unavailable for all infected patients. Accordingly, the Ct-value analyses should be considered exploratory and hypothesis-generating rather than clinically actionable. Fourth, several subgroup analyses—including those according to viral variant, HF phenotype, and GDMT exposure—were limited by relatively small sample sizes and should therefore be interpreted as exploratory. Furthermore, pre-admission HF medication exposure was based on retrospective clinical documentation; medication adherence was not independently adjudicated, and initiation, discontinuation, dose titration, or optimization of GDMT during the index hospitalization could not be systematically assessed. Although the models included measured markers of baseline HF severity, the retrospective data did not allow the contributions of cardiac decompensation and concomitant COVID-19–related systemic or respiratory illness to the observed outcomes to be fully separated. Information on the use of immunosuppressive agents was not systematically available and therefore could not be incorporated into the analyses. Although peak creatinine and nadir eGFR were available as markers of renal dysfunction, detailed temporally adjudicated data required for standardized assessment of acute kidney injury were not systematically available, limiting further evaluation of its contribution to the observed outcomes. Finally, vaccination status was available only for a subset of patients, and exact vaccination dates and the interval since the last vaccine dose were not systematically available. Prior SARS-CoV-2 infection could not be reliably ascertained across the entire cohort. These limitations precluded robust adjustment for individual-level immunity and are particularly relevant when interpreting temporal and variant-specific comparisons. In addition, standardized measures of frailty, general functional status, oxygen requirement, and quantitative severity of respiratory involvement were not systematically available. These unmeasured factors may have contributed to residual confounding and may limit causal interpretation of the observed associations.

### Conclusions

Among patients hospitalized with ADHF, concomitant SARS-CoV-2 infection was associated with substantially worse in-hospital outcomes, including higher rates of in-hospital mortality, ICU admission, invasive mechanical ventilation, as well as longer hospital stays. Higher viral burden was associated with adverse outcomes among infected patients, whereas neither HF phenotype nor the overall burden of pre-existing HF GDMT appeared to be major independent determinants of short-term prognosis. These findings identify ADHF patients with SARS-CoV-2 infection as a particularly high-risk inpatient population and support intensified risk stratification and close multidisciplinary management in this setting. Prospective multicenter studies are warranted to confirm these findings and further refine risk stratification in this vulnerable population. 

## Supplementary Information

Below is the link to the electronic supplementary material.

Supplementary Material 1

## Data Availability

Data supporting the findings of this study can be obtained from the corresponding author upon reasonable request and in accordance with institutional and ethical regulations.

## Abbreviations

ACEi: Angiotensin—converting enzyme inhibitor
ADHF: Acute decompensated heart failure
ALP: Alkaline phosphatase
ALT: Alanine aminotransferase
ARB: Angiotensin receptor blocker
ARNI: Angiotensin receptor—neprilysin inhibitor
AST: Aspartate aminotransferase
BMI: Body mass index
CAD: Coronary artery disease
CK: Creatine kinase
CK-MB: Creatine kinase myocardial band
CKD: Chronic kidney disease
COPD: Chronic obstructive pulmonary disease
CRP: C-reactive protein
Ct: Cycle threshold
eGFR: estimated glomerular filtration rate
GDMT: Guideline—directed medical therapy
GGT: Gamma—glutamyl transferase
GLDH: Glutamate dehydrogenase
HF: Heart failure
HFrEF: Heart failure with reduced ejection fraction
HFmrEF: Heart failure with mildly reduced ejection fraction
HFpEF: Heart failure with preserved ejection fraction
ICU: Intensive care unit IQR—interquartile range
LDH: Lactate dehydrogenase
LVEF: Left ventricular ejection fraction
MRA: Mineralocorticoid receptor antagonist
NT-proBNP: N-terminal pro-B-type natriuretic peptide
NYHA: New York heart association
OR: Odds ratio
RSV: Respiratory syncytial virus
RT-qPCR: Reverse transcription quantitative polymerase chain reaction
SARS-CoV-2: Severe acute respiratory syndrome coronavirus 2
SGLT2i: Sodium-glucose cotransporter-2 inhibitor
sPAP: systolic pulmonary artery pressure
TAPSE: Tricuspid annular plane systolic excursion
