# Supplementary material for: Impact of SARS-CoV-2 infection and viral burden on outcomes in acute decompensated heart failure
Source: BMC Infect Dis. 2026 Sep 18;26:1694. doi: 10.1186/s12879-026-14407-y (PMC13595687; doi:10.1186/s12879-026-14407-y)
Supplement: Supplementary file 1 [file 12879_2026_14407_MOESM1_ESM.docx]

**Supplementary Table 1.** **Documented causes of death according to SARS-CoV-2 infection status**

| **Cause of death** | **Total (n=441)** | **SARS-CoV-2 positive (n=80)** | **SARS-CoV-2 negative (n=361)** | ***p*-value** |
| --- | --- | --- | --- | --- |
| Pneumonia | 114 (25.9) | 34 (42.5) | 80 (22.2) | **0.0002** |
| Heart failure | 96 (21.8) | 24 (30.0) | 72 (19.9) | **0.0486** |
| Malignancy | 72 (16.3) | 6 (7.5) | 66 (18.3) | **0.0182** |
| Other cardiovascular/thromboembolic causes | 69 (15.6) | 9 (11.3) | 60 (16.6) | 0.2316 |
| Cerebrovascular/neurologic causes | 51 (11.6) | 5 (6.3) | 46 (12.7) | 0.1004 |
| Other causes | 39 (8.8) | 2 (2.5) | 37 (10.2) | **0.0272** |

**Supplementary Table 1.** Documented causes of death according to SARS-CoV-2 infection status. Data are presented as n (%), with percentages calculated within the respective groups of patients who died during the index hospitalization. Causes of death were determined from the available clinical records and grouped into mutually exclusive clinically relevant categories. Other cardiovascular/thromboembolic causes comprised cardiovascular causes other than heart failure, including thromboembolic causes. Other causes comprised gastrointestinal, hepatic, renal, metabolic, and trauma-related causes. Between-group comparisons were performed using the Pearson χ² test.

**Abbreviation:** SARS-CoV-2, severe acute respiratory syndrome coronavirus 2.

**Supplementary Table 2. Univariable and multivariable regression analyses of the association between SARS-CoV-2 infection and in-hospital outcomes in patients hospitalized with acute decompensated heart failure.**

| **Outcome** | **Univariable effect estimate** | **Univariable 95% CI** | **Univariable p-value** | **Multivariable effect estimate** | **Multivariable 95% CI** | **Multivariable**  ***p*-value** |
| --- | --- | --- | --- | --- | --- | --- |
| ICU admission | OR 1.54 | 1.18–2.03 | **0.0017** | OR 1.79 | 1.22–2.62 | **0.0027** |
| Invasive mechanical ventilation | OR 2.84 | 1.53–5.03 | **0.0014** | OR 2.84 | 1.51–5.11 | **0.0017** |
| In-hospital mortality | OR 1.86 | 1.39–2.48 | **<0.0001** | OR 2.63 | 1.61–4.21 | **0.0002** |
| Length of hospital stay (days) | β 8.17 | 5.93–10.40 | **<0.0001** | β 9.23 | 6.52–11.94 | **<0.0001** |
| ICU length of stay (days) | β 6.58 | 3.24–9.91 | **0.0001** | β 7.85 | 2.97–12.73 | **0.0017** |

**Supplementary Table 2.** Univariable and multivariable regression analyses evaluating the association of SARS-CoV-2 infection status with in-hospital outcomes among patients hospitalized with acute decompensated heart failure. Logistic regression models were used for binary outcomes (ICU admission, invasive mechanical ventilation, and in-hospital mortality), and linear regression models were used for continuous outcomes (length of hospital stay and ICU length of stay). Effect estimates are presented as odds ratios (ORs) or regression coefficients (β) with 95% confidence intervals (CIs). Multivariable models were adjusted as described in the Methods section. For invasive mechanical ventilation, a parsimonious multivariable model was applied because of the limited number of events.

**Abbreviations:** ICU, intensive care unit; SARS-CoV-2, severe acute respiratory syndrome coronavirus 2.

**Supplementary Table 3. Documented infectious complications during the index hospitalization according to SARS-CoV-2 infection status.**

| **Variable** | **Total (n=2002)** | **SARS-CoV-2 positive (n=246)** | **SARS-CoV-2 negative (n=1756)** | ***p*-value** |
| --- | --- | --- | --- | --- |
| Bacterial pneumonia | 465 (23.2) | 63 (25.6) | 402 (22.9) | 0.3446 |
| Urinary tract infection | 81 (4.0) | 15 (6.1) | 66 (3.8) | 0.0812 |
| Bloodstream infection/bacteremia | 112 (5.6) | 20 (8.1) | 92 (5.2) | 0.0646 |
| Sepsis | 387 (19.3) | 58 (23.6) | 329 (18.7) | 0.0717 |

**Supplementary Table 3.** Data are presented as n (%). Between-group comparisons were performed using the χ² test.

**Abbreviations:** SARS-CoV-2, severe acute respiratory syndrome coronavirus 2.

**Supplementary Table 4. Thromboembolic events during the index hospitalization according to SARS-CoV-2 infection status.**

| **Variable** | **Total (n=2002)** | **SARS-CoV-2 positive (n=246)** | **SARS-CoV-2 negative (n=1756)** | ***p*-value** |
| --- | --- | --- | --- | --- |
| Any thromboembolic event | 59 (2.9) | 5 (2.0) | 54 (3.1) | 0.3651 |
| Cerebral infarction due to thrombosis or embolism | 35 (1.7) | 3 (1.2) | 32 (1.8) | 0.7933 |
| Pulmonary embolism | 22 (1.1) | 1 (0.4) | 21 (1.2) | 0.5078 |
| Portal vein thrombosis | 1 (<0.1) | 1 (0.4) | 0 (0.0) | 0.1229 |
| Peripheral arterial embolism/thrombosis | 1 (<0.1) | 0 (0.0) | 1 (0.1) | >0.9999 |

**Supplementary Table 4.** Data are presented as n (%). Between-group comparisons were performed using the χ² test for the composite thromboembolic outcome and Fisher’s exact test for individual diagnoses because of small expected cell frequencies. Among the 22 patients with pulmonary embolism, concomitant deep vein thrombosis was documented in 20; in the remaining two patients, both SARS-CoV-2–negative, no deep vein thrombosis was documented.

**Abbreviation:** SARS-CoV-2, severe acute respiratory syndrome coronavirus 2.

**Supplementary Figure 1.**

**Supplementary Figure 1. Comparison of median SARS-CoV-2 PCR Ct values between survivors and non-survivors among patients with acute decompensated heart failure and SARS-CoV-2 infection.**

Box plots showing the distribution of median SARS-CoV-2 PCR Ct values during hospitalization in survivors and non-survivors. Boxes represent the median and interquartile range, with whiskers indicating minimum and maximum values. Groups were compared using the Mann–Whitney U test. Lower Ct values reflect higher viral load.

**Abbreviations:** Ct, cycle threshold; SARS-CoV-2, severe acute respiratory syndrome coronavirus 2.

**Supplementary Figure 2.**

A

B

C

D

E

**Supplementary Figure 2. Variant-specific differences in in-hospital outcomes among patients with acute decompensated heart failure and SARS-CoV-2 infection.**

Panels illustrate differences in clinical outcomes according to SARS-CoV-2 variant. Categorical outcomes (ICU admission, invasive mechanical ventilation, and in-hospital mortality) are presented as percentages (A–C). Continuous outcomes, including length of hospital stay and ICU length of stay, are shown as box plots displaying the median and interquartile range, with whiskers indicating minimum and maximum values (D–E). The SARS-CoV-2–negative group is included for comparison. Cases with unknown variant status (n = 73) are not shown separately.

**Abbreviations:** ICU, intensive care unit; SARS-CoV-2, severe acute respiratory syndrome coronavirus 2.

**Supplementary Figure 3.**

A

B

C

D

E

**Supplementary Figure 3. Heart failure phenotype–specific differences in in-hospital outcomes among patients with acute decompensated heart failure and SARS-CoV-2 infection.**

Panels illustrate in-hospital outcomes stratified by heart failure phenotype (HFrEF, HFmrEF, and HFpEF). Categorical outcomes (ICU admission, invasive mechanical ventilation, and in-hospital mortality) are presented as percentages (A–C). Continuous outcomes, including length of hospital stay and ICU length of stay, are shown as box plots displaying the median and interquartile range, with whiskers indicating minimum and maximum values (D–E). Cases with unknown heart failure phenotype (n = 113) are not shown separately.

**Abbreviations:** ICU, intensive care unit; HFrEF, heart failure with reduced ejection fraction; HFmrEF, heart failure with mildly reduced ejection fraction; HFpEF, heart failure with preserved ejection fraction; SARS-CoV-2, severe acute respiratory syndrome coronavirus 2.
